# Variation in Follow-Up after Radical Cystectomy for Bladder Cancer—An Inventory Roundtable and Literature Review

**DOI:** 10.3390/jcm13092637

**Published:** 2024-04-30

**Authors:** Roberto Contieri, Renate Pichler, Francesco del Giudice, Gautier Marcq, Andrea Gallioli, Simone Albisinni, Francesco Soria, David d’Andrea, Wojciech Krajewski, Diego M. Carrion, Andrea Mari, Bas W. G. van Rhijn, Marco Moschini, Benjamin Pradere, Laura S. Mertens

**Affiliations:** 1 Department of Urology, The Netherlands Cancer Institute, 1066 CX Amsterdam, The Netherlands; 2 Department of Biomedical Sciences, Humanitas University, Pieve Emanuele, 20090 Milan, Italy; 3 Department of Urology, Comprehensive Cancer Center Innsbruck, Medical University of Innsbruck, 6020 Innsbruck, Austria; 4 Department of Maternal Infant and Urologic Sciences, Sapienza University of Rome, Policlinico Umberto I Hospital, 00185 Rome, Italy; 5 Urology Department, Claude Huriez Hospital, CHU Lille, F-59000 Lille, France; 6 Department of Urology, Fundació Puigvert, Autonomous University of Barcelona, 08193 Barcelona, Spain; 7 Urology Unit, Department of Surgical Sciences, Tor Vergata University Hospital, University of Rome Tor Vergata, 00133 Rome, Italy; 8 Division of Urology, Department of Surgical Sciences, AOU Città della Salute e della Scienza di Torino, Torino School of Medicine, 10126 Torino, Italy; 9 Department of Urology, Medical University of Vienna, 1090 Vienna, Austria; david.dandrea@meduniwien.ac.at; 10 Department of Minimally Invasive Robotic Urology Center of Excellence in Urology, Wrocław Medical University, 50-556 Wroclaw, Poland; 11 Department of Urology, Torrejon University Hospital, 28850 Madrid, Spain; 12 Oncologic Minimally Invasive Urology and Andrology Unit, Department of Experimental and Clinical Medicine, Careggi Hospital, University of Florence, 50121 Florence, Italy; 13 European Association of Urology, Non-Muscle Invasive Bladder Cancer Guidelines Panel, 6803 AA Arnhem, The Netherlands; 14 Department of Urology, IRCCS San Raffaele Hospital, Vita-Salute San Raffaele University, 20132 Milan, Italy; marcomoschini87@gmail.com; 15 Department of Urology, Comprehensive Cancer Center, Medical University of Vienna, 1090 Vienna, Austria; 16 Department of Urology UROSUD, La Croix Du Sud Hospital, F-31130 Quint-Fonsegrives, France

**Keywords:** follow-up, radical cystectomy, bladder cancer

## Abstract

**Background:** Follow-up after radical cystectomy (RC) for bladder cancer can be divided into oncological and functional surveillance. It remains unclear how follow-up after RC should ideally be scheduled. The aim of this report was to gain insight into the organization of follow-up after RC in Europe, for which we conducted a roundtable inventory within the EAU Young Academic Urologists Urothelial Cancer working group. **Methods:** An inventory semi-structured survey was performed among urologists of the EAU Young Academic Urologists Urothelial Cancer working group to describe the organization of follow-up. The surveys were analyzed using a deductive approach. Similarities and differences in follow-up after RC for bladder cancer were described. **Results:** The survey included 11 urologists from six different European countries. An institutional follow-up scheme was used by six (55%); three (27%) used a national or international guideline, and two (18%) indicated that there was no defined follow-up scheme. Major divergent aspects included the time points of follow-up, the frequency, and the end of follow-up. Six centers (55%) adopted a risk-adapted follow-up approach tailored to (varying) patient and tumor characteristics. Laboratory tests and CT scans were used in all cases; however, the intensity and frequency varied. Functional follow-up overlapped with oncological follow-up in terms of frequency and duration. Patient-reported outcome measures were only used by two (18%) urologists. **Conclusions:** Substantial variability exists across European centers regarding the follow-up after RC for bladder cancer. This highlights the need for an international analysis focusing on its organization and content as well as on opportunities to improve patients’ needs during follow-up after RC.

## 1. Introduction

Radical cystectomy (RC), including bilateral pelvic lymph node dissection (PLND), eventually preceded by neoadjuvant chemotherapy, is the standard treatment for patients with non-metastatic muscle-invasive bladder cancer (BC) or very-high-risk non-muscle invasive BC [1]. 

RC is a major surgical procedure involving the removal of the bladder and surrounding organs, which may include the prostate and seminal vesicles in men or the uterus, ovaries, and part of the vagina in women. Postoperative urinary diversion is necessary due to the removal of the bladder. Options for diversion include an ileal conduit, in which the ureters are attached to a segment of the ileum that forms a stoma on the abdominal wall; an orthotopic neobladder constructed from intestinal segments replaces the bladder and allows volitional urination through the urethra. The selection of urinary diversion is influenced by oncologic outcomes, patient anatomy, personal preference, and functional status. Additionally, the tumor may recur locally at the initial site or manifest as distant metastases, necessitating systematic and periodic evaluations over time to ensure early detection and intervention.

Therefore, follow-up after RC can be divided into oncological and functional surveillance. The goal of oncological follow-up is to detect disease recurrence, whereas functional follow-up focuses on urinary, sexual, renal, and metabolic function and on quality of life. 

BC poses significant challenges due to its propensity for rapid progression and metastasis. These traits underscore the critical need for an intricately tailored and thorough approach to monitoring and managing patients in the post-treatment phase. The complexity of BC’s clinical behavior demands not only vigilant surveillance to detect recurrences at an early stage but also a nuanced understanding of the individual patient’s clinical profile to devise a personalized management plan. This approach facilitates the optimization of patient outcomes, mitigating the risk of recurrence and improving quality of life through timely intervention and tailored therapeutic strategies.

To date, the scheduling of follow-up care after RC remains a topic of ambiguity, with international guidelines offering no explicit recommendations for its optimal timing or frequency [1,2]. This absence of standardized guidance contributes to a substantial heterogeneity in follow-up practices across clinical settings. Our hypothesis posits that this variability in post-RC follow-up protocols significantly influences patient outcomes and management strategies, suggesting a critical need for empirical investigation to establish evidence-based guidelines.

In the current study, we sought to systematically investigate and evaluate the prevailing follow-up protocols implemented after RC among leading European BC experts. Therefore, we conducted a survey among the EAU Young Academic Urologists (YAU) Urothelial Cancer working group to identify similarities and differences in adopted follow-up schemes after RC. This initiative was designed to identify both the convergences and divergences in the follow-up schemes adopted after RC, thereby shedding light on the spectrum of clinical approaches within European cancer centers and analyzing them in the context of the available scientific literature and evidence base. Furthermore, the existing literature predominantly concentrates on the efficacy of treatments while frequently neglecting the structure, content, and intensity of follow-up. Therefore, to comprehensively contextualize our findings within the existing body of research, a narrative synthesis of relevant literature was conducted. The ultimate goal was to gather insights that could potentially inform the development of more standardized and evidence-based follow-up protocols, enhancing patient care and outcomes in the post-operative setting.

## 2. Materials and Methods

This study used an explorative design, including semi-structured interviews with 11 urologists who were part of the YAU Urothelial Cancer working group. All respondents were aware of the aim of the study, and no patients or patient data were involved. The respondents were asked to provide a detailed description of the institutional follow-up pathway (i.e., a care pathway defined for a specific hospital) for BC patients after RC. In case there was no predefined follow-up pathway, respondents were asked to describe how follow-up was carried out. Eleven sub-questions were asked to guide the respondents’ answers and ensure all follow-up aspects were covered (Supplementary). As follow-up schedules may vary depending on several factors, including the final pathology staging, lymph node involvement, the type of urinary diversion, and the use of adjuvant chemotherapy, respondents were asked to describe these specific points. The questions were not closed-ended, and no predefined answers were used to provide comprehensive qualitative insights.

The data obtained from the surveys were analyzed using a deductive approach to describe the follow-up after RC. The variation in the follow-up organization was objectively assessed in terms of timing, frequency, follow-up methods (physical examination, laboratory analysis, imaging, and the disciplines of the involved healthcare professionals. Similarities and differences in the organization of follow-up were then described.

The primary aim of this study was to document the direct experiences of some of the largest oncological centers in Europe, providing an invaluable perspective on the variations and commonalities in post-RC follow-up protocols across different healthcare settings.

We performed a narrative synthesis of the literature. An extensive search was conducted across multiple databases, including PubMed, Scopus, and Web of Science, using a combination of keywords and MeSH terms such as “Radical cystectomy,” “urothelial carcinoma,” “muscle invasive bladder cancer”, “Follow-up”, and “recurrence after Radical cystectomy”. The inclusion criteria were studies that provided significant insights about the follow-up of patients after radical cystectomy. The selected articles were then reviewed for their relevance, rigor, and contribution to the field of urologic oncology, with a focus on identifying gaps in current knowledge and potential directions for future research. A narrative synthesis of these data was then undertaken.

## 3. Results

The study included 11 participants; all of them were under 40 years of age and had completed their residency or fellowship within the past five years. The participants were practicing urologists in European referral centers (France (*n* = 2), Italy (*n* = 3), Spain (*n* = 2), Austria (*n* = 2), Poland (*n* = 1), and the Netherlands (*n* = 1)). Six urologists (55%) indicated that an institutional follow-up scheme was used, two urologists (18%) indicated that they used national guidelines, and one (9%) followed “European guidelines”. Two others (18%) indicated that there was no defined follow-up scheme.

**Figure 1:**
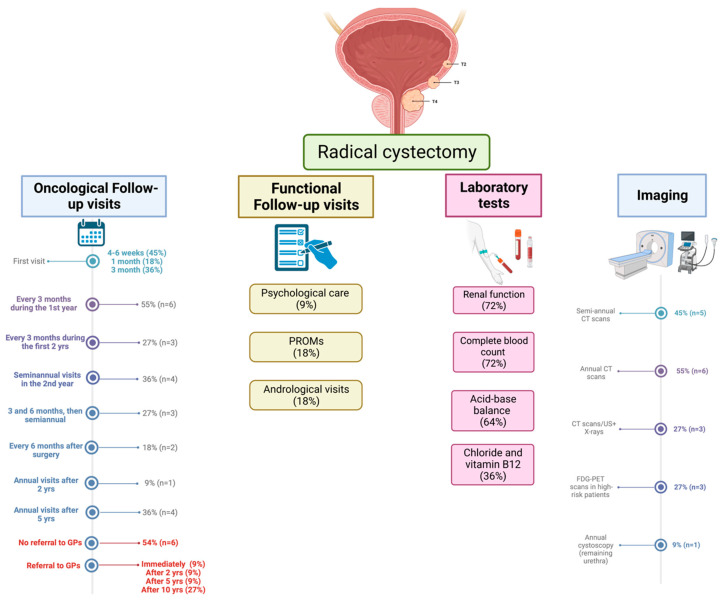
Summary of the results of the semi-structured interviews.

The results are summarized in Figure 1.

Most urologists scheduled the first follow-up visit after RC between four to six weeks post-surgery (*n* = 5; 45%) or three months post-surgery (*n* = 4; 36%). In two cases (18%), the first follow-up visit was scheduled within the first month after RC. Six urologists (55%) scheduled follow-up visits every three months during the first year after surgery, followed by semiannual visits in four cases, while three continued trimestral visits for up to two years. Three urologists (27%) scheduled follow-up visits three and six months after surgery and then semiannual visits. In two cases (18%), follow-up visits were scheduled every six months from the start. At four hospitals (36%), follow-up visits were further de-intensified to annual visits after five years, while in one hospital (9%), this occurred after two years.

Six respondents (54%) indicated utilizing a risk-adapted follow-up tailored according to patient characteristics. For instance, at one center, patients were stratified into high- and low-risk groups according to their pathological T-stage (>pT2), pathological N-stage (N+), and the presence of lymphovascular invasion (LVI). Patients with at least one of these factors present were classified as high-risk. During the first five years of follow-up, low-risk patients underwent a lower number of computed tomography (CT) scans (5 versus 7) and a higher number of ultrasound examinations (8 versus 6) compared with high-risk patients.

In all centers, laboratory tests were performed along with follow-up visits. Laboratory tests included renal function and complete blood count (72%). The acid–base balance was measured routinely by seven urologists (64%). Five urologists (46%) reported not measuring chloride and vitamin B12 regularly, while this was only performed in patients with a neobladder, according to two urologists (18%).

Computed tomography (CT) was the most commonly used imaging technique for follow-up. The intensity and frequency of imaging varied: five urologists (45%) conducted semi-annual CT scans, whereas others (55%) conducted annual CT scans. According to three urologists (27%), CT scans were alternated with ultrasound and chest X-rays. Positron emission tomography (PET) scans with fluorodeoxyglucose (FDG) were used for selected high-risk patients by three urologists (27%). One urologist (9%) performed annual cystoscopies in case of preservation of the urethra.

The end of oncology follow-up was scheduled at five and ten years, according to two (18%) and three (27%) of the urologists, respectively. at five centers (45%), there was no end of oncological follow-up visits, which continued lifelong. The referral of RC patients to general practitioners (GPs) occurred immediately after surgery in one case (9%) and after two years (9%), after five years (9%), or after 10 years (27%) post-surgery. At six centers (54%), there were no referrals to GPs.

Follow-up visits routinely included questions related to functional items at all centers. One urologist (9%) reported that his medical center offered psychological care to all patients undergoing RC; two (18%) reported that an andrologist was involved in patient follow-up upon request. Only two urologists (18%) routinely used patient-reported outcome measures (PROMs) in functional follow-ups. The duration of functional follow-up overlapped with oncologic follow-up in nine cases (82%).

## 4. Discussion

In the present report, we described the results of an inventory roundtable among eleven European urologists. We found considerable variability in the frequency, content, and organization of follow-up after RC. Major divergent aspects included the timing of follow-up, the frequency, and the end of follow-up. The optimal approach for follow-up after RC for bladder cancer remains unclear.

First, there is no uniform use of a guideline for follow-up. Indeed, only 27% used a national or European follow-up schedule; this highlights the lack of a well-defined European scheme. For example, the EAU guideline on muscle-invasive and metastatic bladder cancer gives general advice, such as “after radical cystectomy with curative intent, regular follow-up is needed” [3], but no defined schedule [1] as, for example, in non-muscle invasive bladder cancer [4] or renal cell carcinoma [5].

Initial follow-up visits were typically scheduled between four weeks to six months post-surgery. Follow-up protocols varied, with six centers adopting risk-adapted strategies based on prognostic factors. Routine follow-ups included comprehensive lab tests and varied imaging schedules, primarily using CT scans. Interestingly, one center incorporated psychological support and, surprisingly, only two centers adopted PROMs.

The absence of clear guidance on follow-up regimens should be attributed to the limited availability of high-quality evidence. Indeed, the studies investigating this topic are retrospective and employ varying follow-up regimens and imaging techniques, thus limiting the ability to establish consistent guidelines [6,7,8]. As a result, many practitioners appear to need to set up their own follow-up schedules, which are primarily based on local regulations and personal experience.

Second, the lack of precise guidelines contributes to the wide variation in the timing, frequency, and format of follow-up. According to a recent consensus, CT scans are recommended up to five years postoperatively [3]; however, the optimal frequency has yet to be defined. The frequency of follow-up, however, is a critical issue, as follow-up puts a strain on healthcare resources and costs, while at the same time, the effect of follow-up on oncologic outcomes needs to be clarified.

The incidence of local recurrence post-RC varies between 5% and 15%, while systemic recurrence can be as high as 50% [9]. Recurrences are most likely to occur within 36 months following RC, with the highest concentration observed between 6 and 18 months [9]. Recurrences in the urethra are less common (4–6%) and tend to present within the first three years [10]. Upper tract urothelial carcinoma has an estimated incidence rate of 4% to 10%. Typically, these tumors develop later, with 70% of cases observed three years after surgery [11]. All in all, recurrences are mainly diagnosed during the first three years post-surgery, with a peak incidence between 6 and 18 months [9]. Although it seems essential to be alert for the first years after RC, there are no prospective studies demonstrating a survival advantage in early detection of recurrence compared with detection at the time of symptom occurrence [1]. In addition, the results of published retrospective series on this topic are based on applying different follow-up schemes and imaging techniques and provide conflicting results [6,7,8].

Furthermore, according to multiple authors, when providing individual recommendations for follow-up, the risk of competing health factors must also be considered. Indeed, Stewart-Merril et al. found that in elderly, low-risk patients, the risk of non-bladder cancer-related mortality was consistently higher than the risk of recurrence, suggesting that follow-up can be stopped early in these patients [12]. Recently, Martini et al. proposed a similar scheme considering patients with histological variants [13]. This illustrates that there is room to optimize (risk-adjusted) follow-up.

Factors such as tumor and nodal stage, subtype histology molecular profile, and age should be taken into account to determine the optimal frequency and intensity of surveillance. This approach recognizes that not all patients have the same level of risk and ensures that resources are allocated appropriately to those who need them the most.

Functional outcomes following RC should not be underestimated. Urinary diversion-related complications can be seen in up to 45% within the first five years of follow-up. This risk increases to 94% in patients surviving beyond 15 years [14]. Functional complications include but are not limited to vitamin B12 deficiency [15], metabolic acidosis, renal function deterioration, urinary infections, urolithiasis, herniation or stenosis of the stoma, and ureteroenteric strictures. Furthermore, patients with intestinal urinary diversion seem to be at higher risk of bone fractures [16]. For example, Nieuwenhuijzen et al. found a low level of B12 vitamin in 17% of patients with bowel diversion; therefore, the EAU guidelines recommend annual measurement of vitamin B12 levels in patients with this type of diversion [1].

Patients with neobladders often experience continence problems and emptying dysfunction [10], with approximately two-thirds of women requiring catheterization of their neobladder, and almost 45% are unable to void spontaneously [17]. In a retrospective study, ileal conduits were found to have fewer late complications compared with continent abdominal pouches or orthotopic neobladders [15]. However, good functional outcomes can be achieved at high-volume centers with dedicated teams [18]. Thus, in addition to oncological follow-up, it is essential to implement regular functional evaluations to ensure the early detection and management of complications and to optimize patient outcomes following RC. It is critical to emphasize the importance of proper functional follow-up in the management of these patients, who are often frail and require careful monitoring and follow-up care to ensure optimal outcomes.

In the absence of high-level evidence, some follow-up patterns have been proposed on the basis of the natural history of the disease and expert opinion. In an effort to provide clear and shared expert guidance, the European Association of Urology (EAU) and the European Society for Medical Oncology (ESMO) collaborated to create a consensus statement on controversial topics in bladder cancer management. The experts agreed that regular follow-up is necessary for patients after RC and that CT scans of the abdomen and chest should be performed for the first five years to detect relapse after radical treatment. However, the proposed follow-up schedule, which recommended monitoring every 3–4 months during the first two years, semiannual follow-up for up to 5 years, and yearly thereafter, failed to achieve consensus among experts [3].

The strengths of this study include the use of semi-structured interviews. This enabled urologists to give a comprehensive description of the follow-up practice. However, in a few cases, it may have limited our ability to capture the full spectrum of follow-up practices. An additional limitation is the limited group of selected urologists with a special interest in bladder cancer. Moreover, all participants were younger than 40 years old and were at a relatively early career stage, and therefore, they may have lacked significant personal clinical experience. Importantly, the study only involved urologists from European centers, which may not reflect follow-up practices in other regions globally. However, it is worth noting that all the urologists interviewed were working in referral high-volume centers in Europe. This observation suggests that if substantial variations exist at this level, it is probable that the disparities in follow-up are even more pronounced when examined on a broader scale. Lastly, the interpretations of survey questions by respondents could vary, leading to inconsistencies in how different practices are reported and analyzed. This variability can complicate the comparison of follow-up protocols across centers.

Despite these limitations, this study generates valuable insights into the variation in the follow-up after RC and the unmet need to develop a best-practice follow-up strategy after RC. Ideally, such a scheme should consider the timing and location of local and distant recurrences, the decreasing risk of recurrence over time, and the increasing risk of death from non-BC-related causes [19]. Such a strategy may be tailored to individual patient risk factors. Also, functional follow-up may be optimized. PROMs may be valuable tools in assessing patient-reported functional status. Also, it will be essential to maximize patient engagement, involve patients in the decision-making process, and consider their preferences and expectations when designing follow-up strategies [20]. Indeed, evidence suggests that patient-led and individually tailored follow-up is acceptable and does not compromise outcomes [21].

Additionally, the psychological and emotional aspects should be included, as living with an aggressive disease can be mentally challenging. By incorporating psychosocial support, counseling, and patient education programs into the follow-up protocol, the overall well-being of individuals dealing with bladder cancer can be improved.

## 5. Conclusions

In conclusion, the results of this inventory round table showed that there is substantial variation in follow-up practices after RC among selected European urologists. Furthermore, the narrative review of the literature has illuminated these discrepancies and further substantiated the imperative for standardized follow-up protocols.

This highlights the unmet need for an international analysis focusing on its schedule and content as well as opportunities to improve patients’ needs during follow-up after RC.

## Data Availability

The raw data supporting the conclusions of this article will be made available by the authors on request.
